# Postoperative circulating tumor DNA detection is associated with the risk of recurrence in patients resected for a stage II colorectal cancer

**DOI:** 10.3389/fonc.2022.973167

**Published:** 2022-11-10

**Authors:** Adrien Grancher, Ludivine Beaussire, Sylvain Manfredi, Karine Le Malicot, Marie Dutherage, Vincent Verdier, Claire Mulot, Olivier Bouché, Jean-Marc Phelip, Charles-Briac Levaché, Philippe Deguiral, Sophie Coutant, David Sefrioui, Jean-François Emile, Pierre Laurent-Puig, Frédéric Bibeau, Pierre Michel, Nasrin Sarafan-Vasseur, Côme Lepage, Frederic Di Fiore

**Affiliations:** ^1^ Normandie Univ, UNIROUEN, Inserm U1245, IRON group, Rouen University Hospital, Normandy Centre for Genomic and Personalized Medicine and Department of Hepatogastroenterology, Rouen, France; ^2^ Department of Medical Oncology, Henri Becquerel Centre, Rouen, Rouen, France; ^3^ Burgundy Digestive Cancer Registry, INSERM, Lipides, Nutrition, Cancers (LNC)-UMR1231, University Bourgogne Franche-Comté, Dijon, France; ^4^ Paris University, Biology Resources Center EPIGENETEC, Paris, France; ^5^ Department of Digestive Oncology, University Hospital of Reims, Reims, France; ^6^ Department of Gastroenterology and Digestive Oncology, University Hospital of Saint Etienne, Saint Etienne, France; ^7^ Department of Radiotherapy and Medical Oncology, Polyclinique Francheville, Périgueux, France; ^8^ Department of Gastroenterology, St Nazaire Hospital, Saint-Nazaire, France; ^9^ Department of Pathology, Hôpital Ambroise-Paré, Boulogne-Billancourt, France; ^10^ Department of Biology, Georges Pompidou Hospital, Assistance Publique des Hôpitaux de Paris (APHP), Paris, France; ^11^ Department of Pathology, Caen University Hospital, Caen, France

**Keywords:** circulating tumor DNA, liquid biopsy, colorectal cancer, stage II, next generation sequencing

## Abstract

Circulating tumor DNA (ctDNA) is reported to be promising in localized colorectal cancer (CRC). The present study aimed to retrospectively evaluate the impact of ctDNA in patients with a resected stage II CRC from the PROGIGE 13 trial with available paired tumor and blood samples. A group of recurrent patients were matched one-to-one with nonrecurrent patients according to sex, tumor location, treatment sequence, and blood collection timing. CtDNA was analyzed by digital PCR according to NGS of tumors. Disease-free survival (DFS) and overall survival (OS) were analyzed based on ctDNA, and the risks of recurrence and death were determined. A total of 134 patients were included, with 67 patients in each group. At least one alteration was identified in 115/134 tumors. Postoperative ctDNA was detected in 10/111 (9.0%) informative samples and was detected more frequently in the recurrent group (16.7% versus 1.8%; p = 0.02). The median DFS of ctDNA+ versus ctDNA- patients was 16.8 versus 54 months (p = 0.002), respectively, and the median OS was 51.3 versus 69.5 months (p = 0.03), respectively. CtDNA was associated with recurrence (ORa = 11.13, p = 0.03) and death (HRa = 3.15, p = 0.01). In conclusion, the presence of postoperative ctDNA is associated with both recurrence and survival in stage II CRC.

## Introduction

Colorectal cancer (CRC) represents the fourth most common cancer worldwide with an incidence rate of more than 1.8 million per year and approximately 800,000 related deaths (1). At first presentation, more than half of patients have localized disease, including 25% with stage II disease (2). For patients with stage II CRC, surgery alone has shown a high rate of cure of approximately 80% (3), and the role of adjuvant chemotherapy (ACT) is controversial, with a reported increase in survival below 5% (4, 5). Until now, there has been a consensus to propose an ACT in selected patients regarding the presence of high-risk features (6), such as a T4 tumor (7, 8), resection of fewer than 12 lymph nodes (8, 9), lymphovascular or perineural invasion (7, 8), poor differentiation (8, 10) and emergency surgery (10). However, it has been established that these factors are not yet sufficient to clearly identify high- versus low-risk patients (11–13), indicating that new factors are needed to improve patient decision-making.

In this context, the detection of circulating tumor DNA (ctDNA) may be a marker of choice (14, 15). CtDNA is potentially released in the bloodstream by tumor cells and harbors the same main alterations as tumor tissue. CtDNA has been recognized as biologically relevant to reflect tumor dynamics in many solid tumors, including the detection of minimal residual disease (MRD) after curative treatment (16). In patients with CRC, ctDNA has been widely investigated in the metastatic setting with studies showing that its value and variations may be clinically relevant for prognosis evaluation as well as treatment monitoring (17–22). While ctDNA released in the metastatic setting is frequently observed in approximately 80-90% of patients (17, 23–25), its detection remains scarce and more challenging in earlier stages with a postoperative ctDNA detection rate ranging from 5 to 30% in studies including localized CRC (26–31) and at a rate at 8.7% in the specific subgroup of stage II CRC in the Tie et al. study (32). While postoperative ctDNA detection has been reported to be associated with prognosis in patients treated for localized stage I-III CRC, its interest in patient decision making has been recently highlighted in the phase II randomized Australian DYNAMIC trial showing that its postoperative detection may guide adjuvant chemotherapy without compromising the risk of recurrence as compared to standard of care (32–34).

In this context, we conducted a matched case–control study to evaluate the impact of postoperative ctDNA detection in a set of stage II CRC patients from the prospective PRODIGE13 trial.

## Material and methods

### Patients

The present study was conducted based on the prospective French trial, PRODIGE 13, in which 1,925 patients were randomized from 2009 and 2013 into four arms to compare standard versus intensive monitoring as well as the usefulness of the carcinoembryonic antigen (CEA) value in resected stage II and III CRC. Based on a median follow-up of 6.5 years, the 2^nd^ interim analysis results were recently reported without differences in terms of overall survival (OS) between the different strategies (35).

Patients treated for a stage II CRC with a known recurrence during follow-up and with available match paired tumor and blood sample collected after surgery were included in our study. Each recurrent patient with also available tumor and blood sample was then matched one-to-one with a patient without recurrence according to sex, tumor location, neoadjuvant treatment, ACT and the time of sample collection. Patients receiving ACT and/or preoperative chemoradiotherapy (CRT) for rectal tumors were also eligible. Blood samples were optional in the study and were collected after surgery and the timing was not planned. Each patient gave their written consent, and the study was approved by an ethics committee.

### Detection of somatic mutations in primary tumor tissue

For each patient, DNA was extracted from archived formalin-fixed-paraffin embedded (FFPE) tumor tissue using the Maxwell 16 FFPE Plus Lev DNA Purification Kit^®^ (Promega^®^, Fitchburg, Wisconsin, USA). NGS platform was based on AmpliSeq technology and an ion proton sequencer. Libraries were prepared using the colonlungV2 cancer panel. Clonal amplification and sequencing were done on the Ion Chef System (Ion PI Hi-Q Chef, Ion PI Chip Kit v3) and Ion Torrent Proton sequencer (Life Technologies). Data were analyzed by the Torrent Suite 5.0.4 (Life Technologies) using optimized parameters: minimal depth 300×, detection threshold of 2% and 1% for hotspots. Variant call files from the variant caller were loaded on a galaxy platform and annotated using the Safir2report tool (36). NGS coverage depth data were used to identify gene amplifications (in particular ERBB2) using an algorithm developed in the laboratory based on the identification of outliers from the expected coverage mean + 3 standard deviations and calculated using all of the run data (37). For samples with no detected alteration or with an uninterpretable result, a second analysis using another NGS platform was performed based on a QiaSeq targeted custom panel (Qiagen, Hilden, Germany) with 464 amplicons targeting hotspot mutations in CRC on the *APC, BRAF, KRAS, NRAS, PIK3CA* and *TP53* genes. Library was realized using 100 ng of DNA FFPE repaired with NEBNext^®^ FFPE DNA Repair Mix (New England Biolabs). Each sample had a minimal deep sequencing of 500X. Variants were called and annotated by VaScan2, SnpEFF 4.2 and AlamutBatch 1.9. Variant analysis was realized with different database like COSMIC, Clinvar, cBioportal and IARC TP53 database. Selected variants were those either previously described in CRC or class V pathogenic variants.”

### Detection of postoperative ctDNA

Blood samples were collected in EDTA tubes and then centrifuged at 1500 g for 15 minutes to obtain plasma aliquots, which were stored at -80°C. CtDNA was extracted from 0.85 to 4.3 mL of plasma using the QIAamp^®^ Circulating Nucleic Acid Kit (Qiagen, Hilden, Germany) according to the manufacturer’s instructions. A fluorometric technique was used to quantify ctDNA concentrations using the Quant-iT dsDNA high-sensitivity assay (Invitrogen, Life Technologies, Carlsbad, CA, USA) and a Twinkle LB970 microplate fluorometer (Berthold Technologies, France). Concentration of extracted ctDNA was very low due to low release from localized tumor. As we previously reported, a pre-amplification was performed with 10 ng of ctDNA using a PCR of 6 cycles with Q5 Hot Start High Fidelity DNA Polymerase (NEB) and a primer/probe mixture (Taqman SNP Genotyping Assay, Life Technologies) (38). CtDNA was analyzed by droplet digital PCR (ddPCR) based on a Qx200^®^ ddPCR System (Bio–Rad^®^, Hercules, CA, USA) using 2 µL of preamplification, a very sensitive technique that amplify DNA fragment independently in the droplet with probe Taqman^®^. Quantasoft software was used for profile interpretation. DdPCR on ctDNA targeted one of the main somatic alterations identified in the tumor. In cases with no mutation detected by the NGS method, ctDNA analysis was not performed.

The assays performed for ctDNA detection were considered positive if the amount of ctDNA (variant allelic fraction (VAF)) was exceeded a predefined limit of detection (LOD). The threshold of positivity for each of the assays was determined from ctDNA extracted from 12 healthy plasma controls using the following equation: LOD = mean VAF + 1.645 x standard deviation.

### Statistical analysis

The primary objective was to compare the ctDNA detection rate between the recurrent and nonrecurrent matched groups. The secondary objectives were to analyze characteristics of ctDNA+ and ctDNA- patients and evaluate the impact of ctDNA status on OS and disease-free survival (DFS). Patient data were prospectively monitored by the French Federation of Digestive Oncology (FFCD) for the PRODIGE 13 trial. For the purpose of the study, the group of cases was defined as patients with documented disease recurrence during follow-up and available tumor and plasm samples (recurrent group), and each of them was matched one-to-one with patients without recurrence (nonrecurrent group) according to sex, tumor location, neoadjuvant treatment, ACT and the time of sample collection.

The analysis between two groups was performed using Pearson’s chi-squared test, Yate’s continuity correction of the chi-squared test or Fischer’s exact as appropriate. Quantitative variables were compared using an unpaired Student’s t test with Welch’s correction or a Wilcoxon signed ranks test for paired samples. DFS and OS were analyzed using the Kaplan– Meier method and compared with the log rank test. Multivariate analysis of factors associated with the risk of recurrence and death from any cause was performed using logistic regression and a Cox model, respectively, including variables identified in univariate analysis with a p value ≤ 0.10. All statistical tests were two-sided, and statistical significance was set at 0.05. Statistical analyses were performed using SAS 4.0 (SAS Institute Inc., Cary, NC, USA) and R studio version 1.72 (R Foundation for Statistical Computing, Vienna, Austria).

## Results

### Patient characteristics

**Figure 1:**
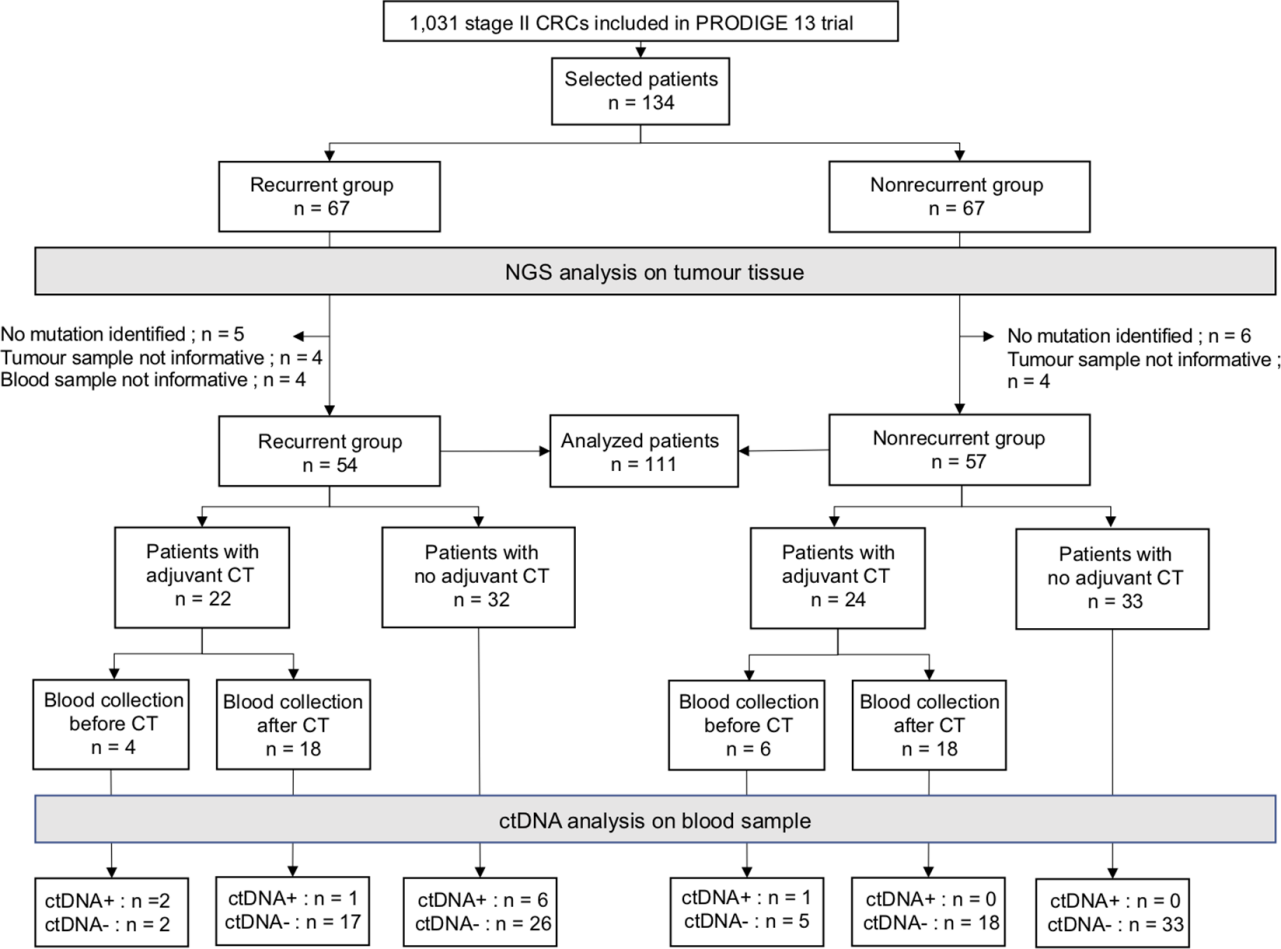
Flow chart of the study population. The overall population consisted of 134 patients with 67 in recurrent and matched nonrecurrent patients. The analysis of ctDNA was based on DNA somatic alterations from tumors detected using two successive NGS panels. At least one alteration was detected in 79/113 patients using the first NGS and in 36/55 remaining patients with the second NGS panel. A total of 115/134 tumors were identified with at least one alteration, corresponding to 58 and 57 patients in the recurrent and nonrecurrent group, respectively. For each patient, ctDNA detection was performed using ddPCR analysis targeting alterations identified with NGS. Timing of blood sample collection was also indicated according to chemotherapy initiation if chemotherapy was used. CRCs, colorectal cancers; CT, chemotherapy; ctDNA, circulating tumor DNA.

A total of 134 stage II patients with available paired blood and tumor samples were included with 67 patients in each of the recurrent and nonrecurrent groups corresponding to 13% of the entire population of stage II from the PRODIGE 13 trial (134/1,045). As shown in **Figure 1**, 126/134 tumors were successfully analyzed by NGS with at least one somatic mutation detected in 115/126 (91.3%). Among the 115 remaining patients, 111 had an informative blood sample, including 54 in the recurrent group and 57 in the matched nonrecurrent group. ACT was performed in 46 patients (41.4%), including 22 (40.7%) and 24 (42.1%) patients in the recurrent and nonrecurrent groups, respectively. For patients with rectal cancer, CRT was performed in 15 cases (60%), corresponding to 10 (76.9%) and 5 (41.7%) patients in the recurrent and nonrecurrent groups, respectively.

The main characteristics of the patients are shown in **Table 1**. As expected, no difference was observed for variables used for matching. Furthermore, no difference was found between the two groups in age, emergency surgery, tumor differentiation or the level of postoperative carcinoembryonic antigen (CEA). Moreover, patients with T4 tumors were more frequent in the recurrent group than in the nonrecurrent group (p=0.04). The median time from surgery to blood collection was 81 and 99 days in the recurrent and nonrecurrent groups, respectively (p=0.53).

**Table 1:** Main characteristics of stage II CRC resected patients included in the study.

Characteristics		Recurrent patients	Nonrecurrent patients	P value	Positive ctDNA	Negative ctDNA	P value
Total		54	57		10	101	
Age	Mean age (years)	66.1	65.9		71.5	65.4	
	Range	38.7 - 85.9	43.3 - 81.3		56.6 - 84.3	38.7 - 85.8	
	<60 years	14 (25.9%)	9 (15.8%)	0.35	2 (20.0%)	21 (20.8%)	**0.04** ^a^
	60-69 years	19 (35.2%)	26 (45.6%)		1 (10.0%)	44 (43.6%)	
	≥70 years	21 (38.9%)	22 (38.6%)		7 (70.0%)	36 (35.6%)	
Sex*	Female	21 (38.9%)	21 (36.8%)	0.82	4 (40.0%)	38 (37.6%)	1^b^
	Male	33 (61.1%)	36 (63.2%)		6 (60.0%)	63 (62.4%)	
Tumor location*	Right-sided	20 (37.0%)	23 (40.4%)	0.91	5 (50.0%)	38 (37.6%)	0.91^a^
	Left-sided	21 (38.9%)	22 (38.6%)		3 (30.0%)	40 (39.6%)	
	Rectal	13 (24.1%)	12 (21%)		2 (20.0%)	23 (22.8%)	
Lymph nodes analyzed	<12	11(20.4%)	11 (19.3%)	0.89	3 (30.0%)	19 (18.8%)	0.41^a^
	≥12	43 (79.6%)	46 (80.7%)		7 (70.0%)	82 (81.2%)	
T stage	T2-3	39 (72.2%)	50 (87.7%)	**0.04**	8 (80.0%)	81 (80.2%)	1^a^
	T4	15 (27.8%)	7 (12.3%)		2 (20.0%)	20 (19.8%)	
VELIPI criteria	None or unknown	34 (70.0%)	40 (70.2%)	0.42	3 (30.0%)	71 (70.3%)	*1* ^a^
	One or more	20 (30.0%)	17 (29.8%)		7 (70.0%)	30 (29.7%)	
Emergency Surgery	No	37 (68.5%)	47 (82.5%)	0.09	6 (60.0%)	78 (77.2%)	0.25^a^
	Occlusion or Perforation	17 (31.5%)	10 (17.5%)		4 (40.0%)	23 (22.8%)	
Differentiation	Well or moderate	49 (90.7%)	50 (87.7%)	0.61	9 (90.0%)	90 (89.1%)	*1* ^a^
	Poor or unknown	5 (9.3%)	7 (12.3%)		1 (10.0%)	11 (10.9%)	
Postoperative CEA	Positive	1 (1.9%)	3 (5.3%)	0.47^a^	7 (70.0%)	94 (93.1%)	**0.047** **^a^**
	Negative	49 (90.7%)	52 (91.2%)		1 (10.0%)	3 (3.0%)	
	Unknown	4 (7.4%)	2 (3.5%)		2 (20.0%)	4 (3.9%)	
Neoadjuvant CRT*	No	44 (81.5%)	52 (91.2%)	0.13	9 (90.0%)	87 (86.1%)	*1* ^a^
	Yes	10 (18.5%)	5 (8.8%)		1 (10.0%)	14 (13.9%)	
Time from surgery to	Median time	81	99	0.53 ^c^	60	91	0.53^d^
blood sample collection (days)	Range	15 - 1,301	29 - 1,842		19 - 1,301	15 - 1,842	
Adjuvant CT*	No	32 (59.3%)	33 (57.9%)	0.88	6 (60.0%)	59 (58.4%)	1^b^
	Yes	22 (40.7%)	24 (42.1%)		4 (40.0%)	42 (41.6%)	
Blood collection after	No	4 (18.2%)	6 (25.0%)	0.84 ^b^	3 (75.0%)	7 (16.7%)	**0.03** ^**a**^
beginning of CT*^§^	Yes	18 (81.8%)	18 (75.0%)		1 (25.0%)	35 (83.3%)	
Postoperative ctDNA status	Negative Positive	45 (83.3%) 9 (16.7%)	56 (98.2%) 1 (1.8%)	**0.02 ^b^**			

*Nonrecurrent patients were initially matched with recurrent patients on these factors. tVELIPI criteria include vascular, lymphatic and/or perineural invasion. ^§^Among patients treated with adjuvant chemotherapy. For p value calculation, Chi-squared test have been used, except if

a Fisher’s exact test for count data.

b Pearson’s Chi-squared test with Yate’s continuity correction.

c Student’s t test or

d Wilcoxon signed ranks test.

e Duration was assessed only on the 36 patients that performed a blood collection after the beginning of adjuvant chemotherapy.

Bold values correspond to p values lower than 0.05.

### CtDNA detection rate and characteristics

Postoperative ctDNA was detected in 10/111 of all patients (9%), and it was significantly more frequent in the recurrent group than in the nonrecurrent group with 9/54 (16.7%) ctDNA+ versus 1/57 (1.8%) ctDNA+ patients (p=0.02). The characteristics of the somatic alterations are listed in **Table 2** for ctDNA+ patients and **Table 1S** for all patients (see **Supplementary Data**). *KRAS* c.35G>A was the most frequent circulating mutation (40%). The variant allele fraction (VAF) was between 0.02% and 15.9% but mostly below 1% (70%), and the median VAF was 0.22% (**Table 2**). Moreover, we analyzed the main somatic mutation detected by NGS in tumor tissue. Comparing ctDNA+ to ctDNA- patients, KRAS mutation was found in 70% vs 42%, BRAF in 20% vs 3%, and TP53 in 10% vs 42%, respectively. Postoperative ctDNA was detected in 14.3% of KRAS-mutated patients, 40% of BRAF-mutated patients, and 2% of TP53-mutated patients (see **Table 2S** in **Supplementary Data**).

**Table 2:** Mutations and variant allele fractions detected in post-operative ctDNA+ patients.

Patient ID	Gene	Mutation	Variant Allele Fraction in tumor (%)	Variant AlleleFraction in ctDNA (%)	Limit of detection of the assay	Time between collection andrecurrence (months)
***Nonrecurrent patients***						
1470	*TP53*	c.524G>A	70.5	0.07	0.055	
***Recurrent patients***						
41	*KRAS*	c.35G>T	60.3	6.8	0.044	0.3
536	*TP53*	c.527G>T	45.6	0.022	0.011	1
886	*KRAS*	c.35G>A	27.3	0.15	0.061	12.6
923	*BRAF*	c.1799T>A	31.9	1.31	0	54.1
1182	*TP53*	c.817C>T	49.1	0.049	0.02	3.3
1496	*KRAS*	c.35G>A	16.8	0.12	0.061	18.2
1573	*KRAS*	c.35G>A	34.4	0.28	0.061	13.4
1857	*KRAS*	c.35G>A	11.4	15.9	0.061	4.2
1905	*BRAF*	c.1799T>A	18.3	0.37	0.048	29.9

As shown in **Table 1**, the presence of postoperative ctDNA was more frequently observed in patients older than 70 years (p=0.03) without a significant difference for other variables, except for postoperative CEA level. No significant difference was found concerning the median time from surgery to blood collection between ctDNA+ (60 days) and ctDNA- patients (91 days) (p=0.53). Among the 10 ctDNA+ patients, the median time between resection and blood sample collection was less than 2 months in 50% of patients, from 2 to 6 months in 20% of patients and more than 6 months in 30% of patients. Among patients treated with ACT (46/111, 41.4%), postoperative ctDNA was more frequently detected when blood samples were collected before (3/10, 30%) than after CT initiation (1/36, 2.8%) (p=0.03). Regarding the 9 ctDNA+ patients from the recurrent group, the median time was 12.8 months from blood collection to clinical recurrence (**Table 2** and **Figure 1S** in **Supplementary Data**).

### CtDNA and prognosis

**Figure 2:**
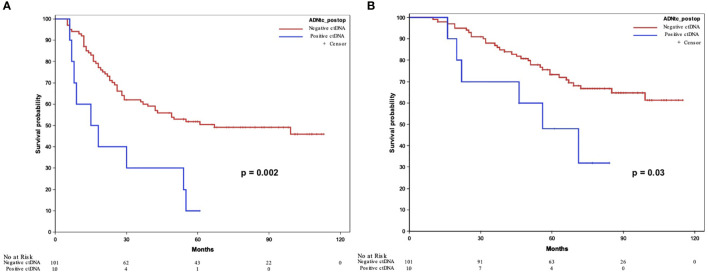
Disease-free survival **(A)** and overall survival **(B)** according to ctDNA detection. ctDNA, circulating tumor DNA.

The presence of postoperative ctDNA was significantly associated with survival in the whole population. The median DFS was 16.8 versus 54 months in ctDNA+ and ctDNA- patients (p=0.002), corresponding to a 3-year DFS of 30% versus 62.1%, respectively. The median OS was 51.3 versus 69.5 months in ctDNA+ and ctDNA- patients, respectively (p=0.03) (**Figures 2A,B**). The presence of postoperative ctDNA was also identified as an independent factor associated with the risk of recurrence in univariate analysis and multivariate analysis [OR=11.20 (95% CI: 1.37-91.71, p=0.02); and adjusted OR (ORa)= 11.13 (95% CI: 1.33-92.91; p=0.03)] (**Table 3**). The presence of postoperative ctDNA was also an independent factor associated with the risk of death in univariate and multivariate analyses [HR = 2.57 (95% CI: 1.07-6.18, p=0.03)] and adjusted HR [HRa = 3.15 (95% CI: 1.28-7.74; p=0.01)] (**Table 4**).

**Table 3:** Logistic regression (univariate and multivariate) assessing the impact of main characteristics on the risk of recurrence (reference: nonrecurrent patients).

Characteristics		Recurrent patients	Nonrecurrent patients	Univariate OR [CI 95%]	P value	Multivariate OR [CI 95%] §	P value
Total		54	57				
Age	<60 years 60-69 years ≥70 years	14 (25.9%) 19 (35.2%) 21 (38.9%)	9 (15.8%) 26 (45.6%) 22 (38.6%)	1 0.47 [0.17-1.31] 0.61 [0.22-1.72]	0.15		
Sex*	Female Male	21 (38.9%) 33 (61.1%)	21 (36.8%) 36 (63.2%)	1 0.92 [0.43-1.97]	0.82		
Tumor location*	Right-sided Left-sided Rectal	20 (37.0%) 21 (38.9%) 13 (24.1%)	23 (40.4%) 22 (38.6%) 12 (21%)	1 1.10 [0.47-2.56] 1.24 [0.47-3.34]	0.66		
Lymph nodes analyzed	<12 >12	11(20.4%) 43 (79.6%)	11 (19.3%) 46 (80.7%)	1 0.93 [0.37-2.38]	0.89		
T stage	T2-3 T4	39 (72.2%) 15 (27.8%)	50 (87.7%) 7 (12.3%)	1 2.75 [1.02-7.39]	**0.045**	1 2.58 [0.91-7.32]	0.07
VELIPI criteria^†^	None or unknown One or more	34 (70.0%) 20 (30.0%)	40 (70.2%) 17 (29.8%)	1 1.12 [0.63-3.06]	0.42		
Emergency surgery	No Yes	37 (68.5%) 17 (31.5%)	47 (82.5%) 10 (17.5%)	1 2.16 [0.89-5.27]	0.09	1 1.61 [0.62-4.24]	0.33
Differentiation	Well or moderate Poor or Unknown	49 (90.7%) 5 (9.3%)	50 (87.7%) 7 (12.3%)	1 0.73 [0.22-2.45]	0.61		
Neoadjuvant CRT*	No Yes	44 (81.5%) 10 (18.5%)	52 (91.2%) 5 (8.8%)	1 2.36 [0.75-7.44]	0.14		
Adjuvant CT*	No Yes	32 (59.3%) 22 (40.7%)	33 (57.9%) 24 (42.1%)	1 0.95 [0.44-2.01]	0.88		
Postoperative ctDNA status	Negative Positive	45 (83.3%) 9 (16.7%)	56 (98.2%) 1 (1.8%)	1 11.20 [1.37-91.71]	**0.02**	1 11.13 [1.33-92.91]	**0.03**

*Nonrecurrent patients were initially matched with recurrent patients on these factors. ^§^ Multivariate regression model includes variables with a p value less than 0.1 in univariate analysis ^†^VELIPI criteria include vascular, lymphatic and/or perineural invasion. CRT, chemoradiotherapy; CT, chemotherapy; ctDNA, circulating tumor DNA.

Bold values correspond to p values lower than 0.05.

**Table 4:** Cox model assessing the impact of postoperative ctDNA status and other characteristics on overall survival.

Characteristics		Univariate Hazard Ratio [CI 95%]	P value	Multivariate Hazard Ratio* [CI 95%]	P value
Age	<60 years 60-69 years ≥70 years	1 0.92 [0.34-2.46] 1.97 [0.80-4.90]	0.14		
Sex	Female Male	1 1.24 [0.64-2.42]	0.53		
Tumor location	Right sided Left-sided Rectal	1 0.79 [0.38-1.66] 1.23 [0.57-2.69]	0.53		
Lymph nodes analyzed	<12 >12	1 0.70 [0.34-1.45]	0.34		
T stage	T2-3 T4	1 1.99 [0.99-4.01]	0.054	1 2.66 [1.26-5.61]	**0.01**
VELIPI criteria	None or unknown One or more	1 1.02 [0.52-1.99]	0.95		
Emergency surgery	No Yes	1 1.31 [0.65-2.63]	0.45		
Differentiation	Well or moderate Poor or unknown	1 2.29 [0.96-5.48]	0.06	1 2.82 [1.15-6.90]	**0.02**
Neoadjuvant CRT	No Yes	1 1.92 [0.88-4.19]	0.10	1 3.12 [1.33-7.34]	**0.01**
Adjuvant CT	No Yes	1 0.87 [0.46-1.67]	0.69		
Postoperative ctDNA status	Negative Positive	1 2.57 [1.07-6.18]	**0.03**	1 3.15 [1.28-7.74]	**0.01**

*Multivariate Cox model contains variables with a p value less than 0.1 in univariate analysis. ^†^VELIPI criteria include vascular, lymphatic and/or perineural invasion. CRT, chemoradiotherapy; CT, chemotherapy; ctDNA, circulating tumor DNA.

Bold values correspond to p values lower than 0.05.

## Discussion

Our results showed that the presence of postoperative ctDNA was significantly associated with the risk of recurrence and shorter DFS and OS in stage II CRC patients. Using a specific ddPCR assay targeting the main alteration detected with NGS methods in tumor DNA, we detected postoperative ctDNA in 9.0% of all patients, and ctDNA was shown to be 9-fold more frequent in the recurrent group than in the nonrecurrent group (16.7% vs. 1.8%, p=0.02). Interestingly, we observed a median time of 12.8 months between ctDNA detection and the diagnosis of disease recurrence. Our results also highlighted that ctDNA status significantly impacted survival with a median DFS of 16.8 versus 54 months (p=0.002) and a median OS of 51.3 versus 69.5 months (p=0.03) in ctDNA+ and ctDNA- patients, respectively. Moreover, the presence of ctDNA was identified as an independent factor associated with the risk of recurrence and death with an adjusted OR of 11.13 (CI 95%:1.33-92.91; p=0.03) and an adjusted HR of 3.15 (CI 95%:1.28-7.74; p=0.01), respectively. Taken together, these results suggested that postoperative ctDNA detection may be a relevant marker to identify high versus low risk of disease recurrence in stage II patients.

The overall rate of postoperative ctDNA+ at 9.0% in our work was similar to previously reported data in stage II and III CRC, ranging from 5.8 to 15%. Indeed, using mixed ddPCR targeting tumor mutations or methylation markers, a recent study that included both stage IIIII patients has reported ctDNA detection at Day 5 after surgery in 6/102 (5.8%) patients in the stage II patient subgroup (39). Using a similar approach to the present study, Tie et al. reported that 20/230 (8.7%) patients were identified as ctDNA positive in their first cohort and 45/299 (15%) in the experimental arm of the DYMAMIC trial (32, 34). In these studies, the rate of postoperative ctDNA was also significantly increased in patients with disease recurrence ranging from 44% to 58%, and in a higher rate than in our work probably due to preanalytical considerations (34, 39). Taken together, these results suggest that postoperative ctDNA is a relatively rare event in localized disease that can be detected in approximately in 10 to 15% of all patients treated for stage II CRC and more frequently in patients with high risk of disease recurrence. However, the exact rate of ctDNA release and its mechanisms need to be further investigated in larger series, including appropriate and dedicated preanalytical processes.

Determining the most appropriate approach for ctDNA detection in early CRC remains challenging, and several factors that may influence the results, such as preanalytical parameters, assay characteristics and the treatment sequence (40). Regarding ctDNA assays, the use of an ultrasensitive method for ctDNA detection, such as a ddPCR assay targeting somatic alterations identified by NGS on tumor DNA, is considered the most efficient. Based on an NGS analysis potentialized with two panels of targeted genes, a somatic alteration was successfully identified in 115/134 (86%) of all tumors, which is close to results reported in the localized CRC stage by Tarazona et al. (132/150, 88%) (27) and Tie et al. (230/250, 92%) (32).

The timing of blood sample collection may also be an important factor to be considered. In our study, ACT was performed in approximately 40% of patients (22/54 in the recurrent group and 24/57 in the nonrecurrent group) on a sample collected after the start of CT in most cases (18/22 and 18/24, respectively). In this setting, we found that ctDNA was more frequently detected before than after ACT initiation (30% vs. 2.8%, respectively) (p=0.03). This finding was in accordance with knowledge about ctDNA kinetics, which is described to change according to treatment sequences, such as from pre- to postsurgery as well as from the start to the end of postoperative CT (31, 41). For example, Chen et al. studied 91 patients treated with perioperative CT and surgery for CRC liver metastasis, and they detected ctDNA in 88% of patients before neoadjuvant CT, in 57% of patients before surgery and in 41% of patients in the postoperative setting (31). Using serial monitoring during ACT in 6 patients with detectable postoperative ctDNA, Tie et al. also showed a complete decrease during the treatment sequence, both in recurrent and nonrecurrent cases. Furthermore, in a study including stage II-III CRC, the detection of ctDNA was observed in 49/178 (27.5%) in the preoperative setting and in 15/171 (10.5%) at Day 5 after surgery, both associated with time to disease recurrence with HRa of 3.58 and 3.22, respectively. Although the best timing for ctDNA detection remains unclear in patients with localized and/or resectable CCR, the postoperative period ranging from 4-8 weeks and the end of ACT are considered the two most relevant time points for the analysis of the prognostic impact of ctDNA detection (29, 31, 32, 34).

We found that ctDNA was found to be significantly associated with DFS, OS and risk of recurrence or death in multivariate analysis. These findings were in accordance with previous data on localized CRC (26, 27, 29, 31) as well as in the only study focusing on stage II (32–34).

This impact on prognosis also translated to the 3-year DFS, which was 30% and 62.1% in the ctDNA+ and ctDNA- patients, respectively, which agreed with the results reported by Tie et al. with a 3-year DFS of 0% and 90%, respectively (32). Interestingly in our work, we also observed that ctDNA detection may precede the diagnosis of disease recurrence using regular follow-up with a median time of 12.8 months, which also agreed with previous findings showing that a ctDNA-positive sample may anticipate imaging evidence of recurrence for at least 3 months (32–35). All these data highlighted that ctDNA is a relevant marker in localized CRC and that its use in routine basis needs to be investigated by prospective trials. In this context, the recent phase II randomized DYMAMIC trial randomized 455 patients to assess whether ctDNA-guided approach could be used for ACT decision without compromising the risk of recurrence as compared to standard of care non ctDNA-guided. The study met its primary endpoint with a non-inferiority in the 2 years recurrence between ctDNA-guided ACT and standard management, respectively 93.5 vs 92.4% (34). Until now, many trials are in progress in localized CRC and the results are awaited to provide additional validation of the ctDNA-guided strategy to individualize adjuvant chemotherapy (42).

The main limitation of the present study was its retrospective design. However, to minimize this limitation, we designed a case–control study with matching according to sex, tumor location, treatment sequence and timing of blood collection. Moreover, these two groups were formed based on the PRODIGE 13 prospective randomized trial and according to the planned prospective follow-up. The other limitations involved the nonstandardized preanalytical process, the presence of only one available and noncodified sample per patient and the use of ACT. Nevertheless, to circumvent these limitations, we specifically designed a molecular assay based on a two-step method using NGS from tumor DNA and dedicated ddPCR for ctDNA detection. Moreover, all these determinant factors involved in the sensitivity of the ctDNA detection rate need to be considered and are now integrated in all ongoing prospective trials. In conclusion, the present study highlighted that the presence of postoperative ctDNA is associated with a higher risk of recurrence and death and shorter DFS and OS in resected stage II CRC.

## Data availability statement

The datasets presented in this study can be found in online repositories. The names of the repository/repositories and accession number(s) can be found in the article/**Supplementary Material**.

## Ethics statement 

This work is an ancillary study from the PRODIGE13 trial, registered on the clinicaltrials.gov website (No. NCT00995202). Each patient included in the PRODIGE13 trial gave written consent. PRODIGE13 was authorized by AFSSAPS (Agence Française de Sécurité Sanitaire des Produits de Santé; No. 2009-A00536-51) on 22/06/2009. The study was performed in accordance with the Declaration of Helsinki. Written informed consent for participation was not required for this study in accordance with the national legislation and the institutional requirements.

## Author contributions

FF, SM, C-BL, KL, PM, and DS designed the study research; OB, J-MP, C-BL, and PD included a large number of patients; LB, CM, SC, J-FE, PL-P, FB, and NS-V performed the molecular analysis; AG, FF, KL, SM, MD, and VV contributed to data analysis and interpretation; and AG, FF, LB, DS, KL, SM, and C-BL wrote the paper. All authors contributed to the article and approved the submitted version.

## Funding

This work was supported by the FARE funding from SNFGE (Société Nationale Française de Gastro-Entérologie).

## Acknowledgments

This study used data collected by the French Digestive Cancerology Federation (FFCD).

## Conflict of interest

The authors declare that the research was conducted in the absence of any commercial or financial relationships that could be construed as a potential conflict of interest.

## Publisher’s note

All claims expressed in this article are solely those of the authors and do not necessarily represent those of their affiliated organizations, or those of the publisher, the editors and the reviewers. Any product that may be evaluated in this article, or claim that may be made by its manufacturer, is not guaranteed or endorsed by the publisher.

## Supplementary material

Supplementary Figure 1 Timeline of patients with positive ctDNA. For recurrent patients, the precise time between blood collection and recurrence is shown in . Blue diamond = date of surgery; red point = date of blood sample; purple arrow = date of recurrence; green square = date of CT starting; blue line = last date of follow-up; and black arrow = date of death.
