# Supplementary figures and images for: Postoperative circulating tumor DNA detection is associated with the risk of recurrence in patients resected for a stage II colorectal cancer
Source: Front Oncol. 2022 Nov 10;12:973167. doi: 10.3389/fonc.2022.973167 (PMC9685416; doi:10.3389/fonc.2022.973167)

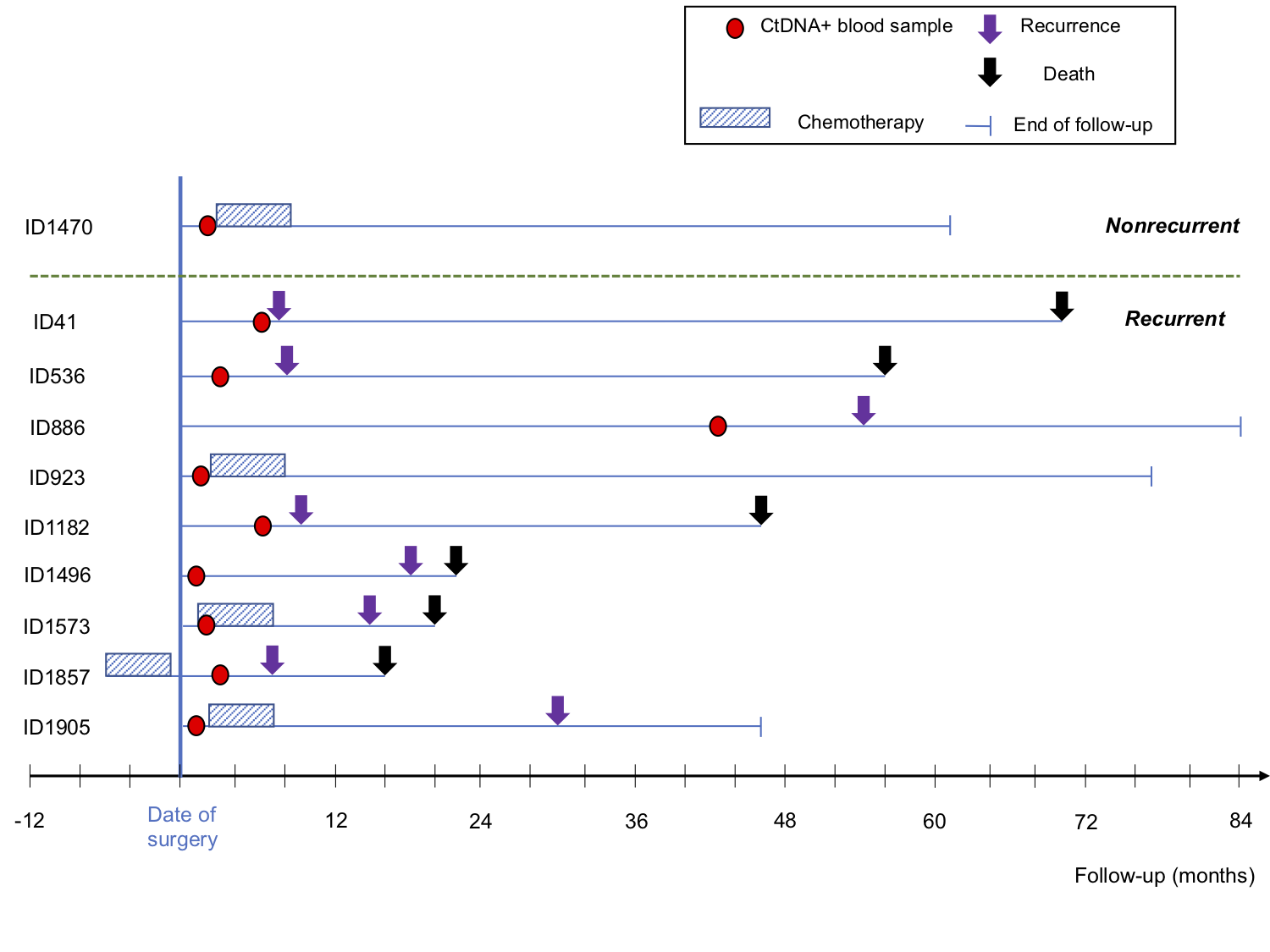

Supplement: Supplementary Figure 1 — Timeline of patients with positive ctDNA. For recurrent patients, the precise time between blood collection and recurrence is shown in . Blue diamond = date of surgery; red point = date of blood sample; purple arrow = date of recurrence; green square = date of CT starting; blue line = last date of follow-up; and black arrow = date of death. [file Image_1.tiff]

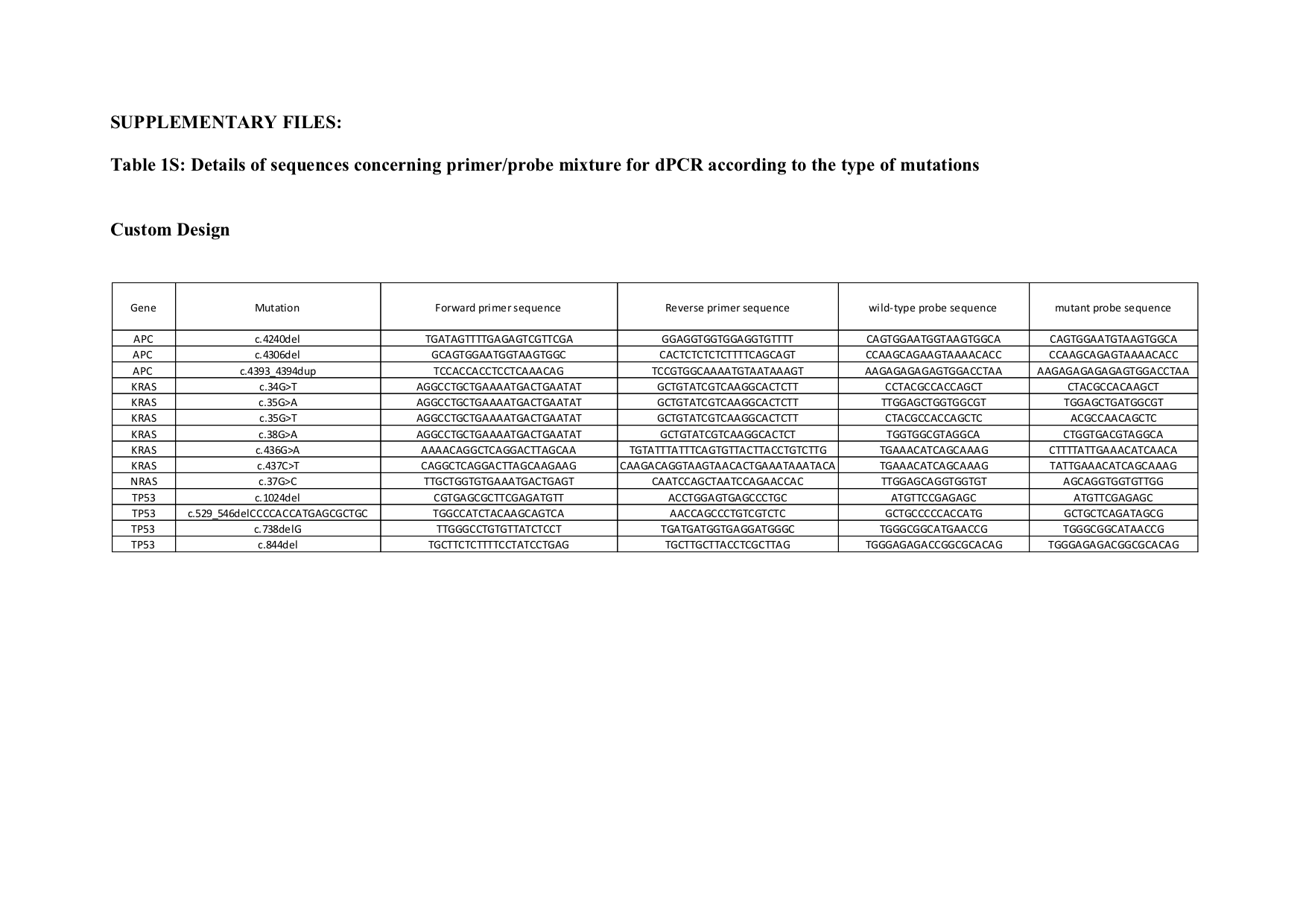

Supplement: Supplementary file 2 [file Image_2.tiff]

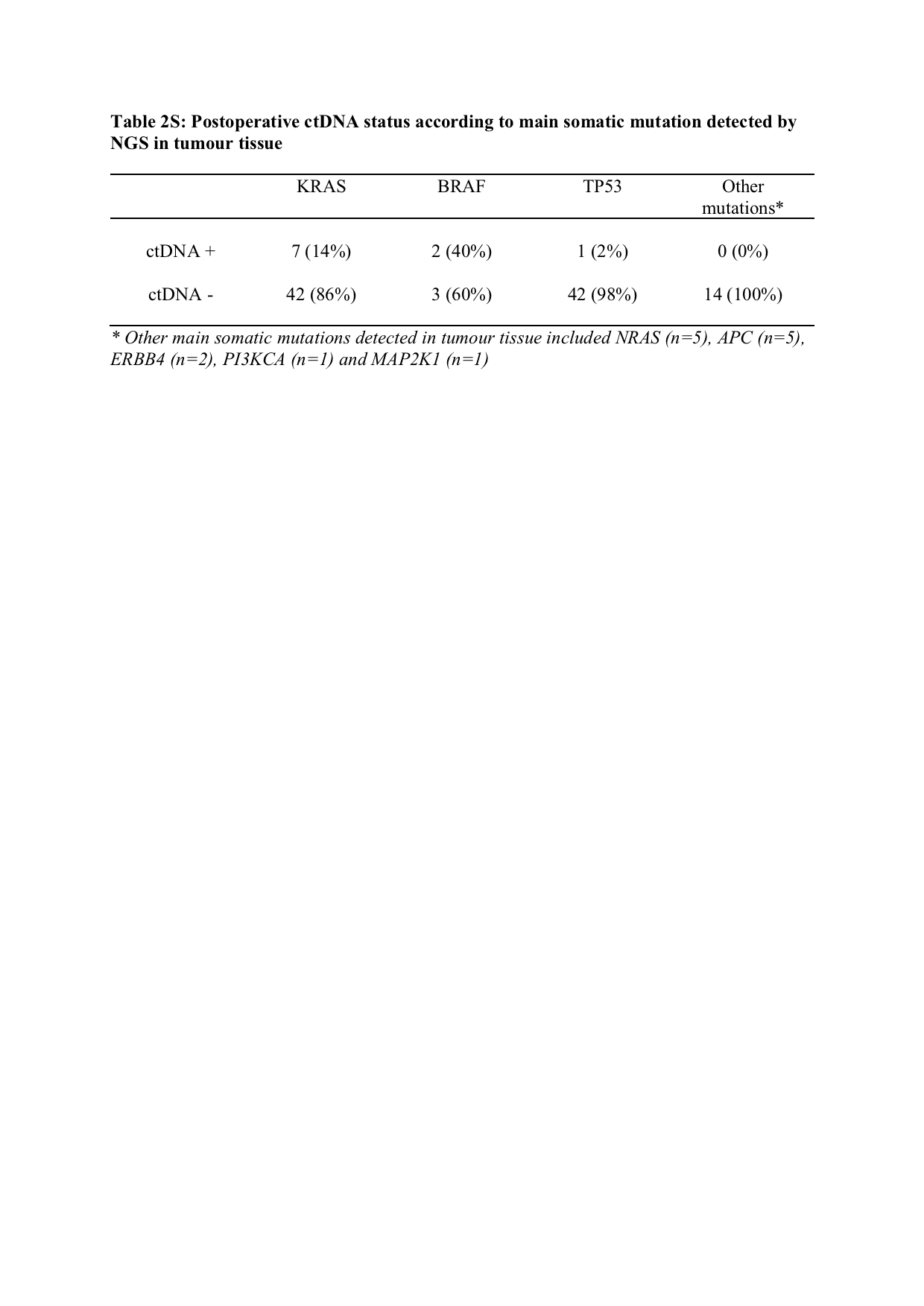

Supplement: Supplementary file 3 [file Image_3.tiff]
